# Eversion of the Uterus in Cows

**Published:** 1898-04

**Authors:** H. P. Keeley

**Affiliations:** Schwenksville, Pa.


### EVERSION OF THE UTERUS IN COWS.

By H. P. Keely, V.M.D.,
SCHWENKSVILLE, PA.

In this paper I do not propose to bring before you anything new or startling; merely to review the causes, symptoms, and treatment, and to tell you what, in my experience, has given good results, knowing that if this paper is discussed I shall learn more than I am able to teach you.

*Definition.* Eversion of the uterus may be defined as the turn-

[1] Read before the annual meeting of the Pennsylvania State Veterinary Medical Association, March, 1898.

ing inside out of the organ—a kind of hernia through the os uteri. It may be turned partial, if it does not protrude beyond the vulva; complete, if it goes beyond the vulva to the exterior of the body, when it usually forms a large, longish tumor, hanging down sometimes as low as the hocks, and showing on its surface the cotyledons, and is usually then accompanied with prolapsus of the floor of the vagina.

*Symptoms.* These are unmistakable. You find a large, longish-shaped tumor coming from between the lips of the vulva. On the surface you will find the cotyledons, and the presence of these makes the diagnosis complete. The floor of the vagina, when prolapsed, is smooth on its surface, and forms more nearly a round or globular tumor, and does not hang down as low. The inverted bladder can be recognized by finding the openings of the ureters and by having the base of the tumor much constricted and issuing from the opening of the urethra. The bag of waters before calving and the fœtal envelopes after calving are not easily mistaken; although I have known it to happen. There is usually not much straining after the eversion is complete, until you come to reduce it. The general symptoms, if any, depend on the length of time since the accident occurred, and are those of pain and uneasiness.

*Complications.* These usually arise from injuries, from neglecting the case by not sending for competent help in time, or allowing ignorant and unskilled persons to attempt reduction, or the animal may injure itself in the stable, or other cattle may tramp on it. Generally there are lacerations, allowing the bladder and intestines to pass out. A case of this kind I saw recently was in a cow not with calf. Before calving she had prolapsus of the vagina and eversion of the uterus after calving. These were treated, and the cow did well, but it was not bred again. About eight or nine months afterward she was found one morning in the stable with a prolapsed vagina which was simply a mass of shreds, the bladder protruded, and about all the small intestine, it appeared to me, was out, hanging away below the hocks. My treatment was to ask the farmer for his rifle to shoot the animal.

*Prognosis.* Depends upon the length of time elapsing between the occurrence of the accident and time of treatment, and upon the condition of the animal. Most cases, if treated promptly, and in a cow of fairly good constitution, not too old or debilitated, will make good recoveries. I have seen cows so weak that they were not able to rise for three or four days make good recoveries. It seems to be a prevalent idea among people that, having once occurred, it is apt to recur every time the cow has a calf. But experience does not bear this out, as cows will frequently, after such an accident, again become pregnant and calve all right without a repetition of the trouble.

*Causes.* Pregnancy must be given a place as a cause, as it never happens except in breeding animals ; in fact, cannot happen except at time of labor, or soon after, when the os is dilated. Difficult labor may cause it at times ; but we all of us have, at times, used much force in extricating the fœtus, and had no eversion, and we know that it happens after the easiest deliveries. Sloping floors have been blamed, but it also occurs on floors that are level. Prolapsus of the vagina during pregnancy, making traction on the uterus and straining its ligaments, has been urged as a cause ; but we know that lots of cows have prolapsus of the vagina during pregnancy, but no eversion of the uterus. It appears that certain predisposing causes are necessary, and then it requires but very little to excite eversion. I believe that we must have a relaxed condition of the ligaments holding the uterus in place. This may be due to a general softness or looseness of the tissues, caused, probably, by soft, sloppy feeding. Given such a condition, it requires only a little turning in of the fundus or one of the cornua, like the end of the finger of a glove, and severe straining, perhaps, from constipation, or excessive peristalsis, or contraction in the womb itself, probably from drinking very cold water—and away it goes, gathering momentum as it goes, until everything is turned inside out. It is a practice among drovers and some farmers to give their cows all the cold water they want to drink immediately after calving, to make them clean, and it often cleans them more thoroughly than they care for ; of course, the owner will never admit anything of this kind.

*Treatment.* Preliminary ; reduction ; retention.

Preliminary : Very often we find our patient down, especially if any considerable time has elapsed since the accident occurred. If possible, get her upon her feet, as it is much more convenient to work at the animal in this position, and the uterus is better retained when once returned, as it goes in on a level or down-hill, instead of being all up-hill work, as it is when the cow is down. If impossible to get her on her feet, we may try to raise the hind part by means of bundles of straw placed under the hips ; but this will usually be found unsatisfactory, and there is then nothing to do but get down on your knees and go to work. Of course, to cleanse the organ of all foreign substances is the first step. If any of the fœtal membranes are still adherent they can easily be removed. A good plan, if you have plenty of help, is to have two men, one on each side, to support the uterus on a clean bag or cloth. If the cow is down, slip a clean bag underneath the uterus for it to rest on. I always order a bucketful of right hot water, to which I put some permanganate of potash with which I bathe the uterus, cleaning it and somewhat reducing the swelling and size. Fleming advises cold water, even pieces of ice, to be rubbed over it; but hot water has always given me good results. Having thoroughly cleansed the uterus, we are ready for the next step.

Reduction: If the everted mass is small, it is an easy matter to return it. But if, as is usually the case, the whole of the uterus is turned out, and the vagina also prolapsed, and has been so for some time, and has become swollen and tumefied, it is certainly no easy job. It is a work requiring lots of patientce, considerable strength, and a great deal of care, lest we injure it by using too much force. Have your two men support the weight of the uterus on a cloth, or bag, while you start to work it in around the edges, a little at a time. Work in one side and hold that within the vulva while you work in a little on the other side, and thus working from side to side, always being on your guard to hold what you have gained against the cow's straining, until you have it pretty well reduced, when you will feel it beginning to go easy. Now you can clap one hand on the remaining portion and shove it in. Having returned the uterus, it is necessary to follow it up with one hand, and smoothe out all the folds, and get the cornua and all the parts to their proper places as nearly as possible. In cases in which the uterus has been out for some time, and has become much swollen, you will find this difficult or impossible, more especially so if the cow is down. But I believe your success will depend, in a great measure, on whether this is done thoroughly or not. You will find your work greatly increased by the animal's straining and working against you. If you can devise means to overcome this straining you will find your work reduced by at least half. Among the methods used to overcome straining is pinching up the skin of the back (farmers sometimes pinch up a fold of the skin and place an awl through it, and a girth around the body or around the chest). Some advise tracheotomy. It seems that this method ought certainly to be effective, because it is impossible for the cow to fix the epiglottis and hold her breath with an opening in the trachea, and without doing this it is impossible for her to strain. I have had no personal experience with tracheotomy in these cases, and I trust that those members who have will favor us with their experience.

Retention : Having returned the uterus and smoothed out all the folds, and everything is in its natural place as nearly as possible, we must devise means for retaining the organ and prevent its being again everted. Among the various means used are pessaries, sutures, skewers, and trusses. The best, I believe, is the pessary, though for the sake of convenience I usually use the sutures. The pessary is best, in my opinion, because it does more to hold the uterus and floor of the vagina to their natural places. The sutures, if properly applied, will not allow the uterus to be protruded to the exterior of the body; but they do not prevent the organ from everting itself and lodging a whole big mass in the posterior portion of the pelvic cavity. I have seen this happen several times, especially in cows too weak to get up. The same is true of skewers and trusses. The pessary, of course, is very difficult or almost impossible to apply when the animal is down.

Pessary : Fleming mentions and describes a number of different kinds of pessaries—the pad, ring, cup and ball, bottle, and pig's bladder. Of these I have had experience with only the first, or pad. It has always given me excellent results. It is a sort of home-made affair, and the materials for making it can generally be found on any farm. To make it, take a short fork- or spade-handle, those with the hand-hold are the most convenient, though this is not strictly necessary, as a hole can be bored to hold your rope, or any round piece of wood of proper thickness and length may be used ; saw it about eighteen inches long ; about four or six inches from one end cut a groove clear around to hold the string with which you tie the pad. Then make the pad on this end, by wrapping it with rags or cloths until you have a pad about the size of two fists, then tie securely with a string in the groove you have cut, and it is ready for use. Before inserting it, I always dip the pad in melted lard. Then insert it into the vagina as far as may be necessary. Now, to hold it in place, take a rope and knot the middle of it to the handle. Take an end up on each side of the tail, on the croup make a single loose knot, go forward along the back to the withers, make another knot down on each side of the neck, knot again back between the front legs, up on each side of the body, pass an end through the rope on each side at the back, go down and back through between the hind legs on each side of the udder and up to your starting place. Tighten your ropes and tie securely. This I believe to be the best method when the cow is able to stand for you to apply it. It makes no difference if she does go down afterward. The other pessaries are applied in practically the same way, and are no doubt good; but having never tried them, I can say nothing about them.

Sutures: These are of two kinds, labial and hip; labial when only the lips of the vulva are sutured, and hip when they are passed through the skin of the hips. My method of suturing is this: Start with a seton-needle and a stout piece of tape; take a hold in the thick skin of the hip on one side a little below the level of the superior commissure of the vulva; go across, taking a deep hold through both lips of the vulva, and on over again through the skin on the hip on the other side; then cross obliquely to the first side, and repeat at about the level of the inferior third of the vulva. Then draw up and tie your two ends securely.

For these two methods, the only ones I ever used, I do not claim any originality. They were taught me by our worthy ex-President, Dr. Ridge.

Trusses: About these and skewers I shall not have much to say, never having used them. But it seems to me that the pessary has the same advantage over these that it has over sutures; it maintains the parts in place, while the trusses and sutures simply prevent the organ from being protruded to the exterior of the body. Before leaving our subject, we must never forget to order the cow so placed that she will stand or lie high behind and low in front.

*After-treatment.* This must be governed by the individual case, and by meeting emergencies as they arise. Keep the cow standing high behind. Avoid constipation. Feed so as to keep the bowels open; give no very cold water. If there be fever, treat accordingly. If there is straining, you may give anodynes, but nothing that will constipate; cannabis indica may be given—its tendency to constipate is not great. We may put a tight girth around the body or chest. The pessary or suture may safely be left as long as deemed necessary, as long as they do not interfere with the natural functions, defecation and micturition.

*Amputation.* About this method of treatment I shall have but little to say. I tried it once—result, dead cow. It happened when I was in that stage of my professional career when a man is ready to do or try anything that the owner will permit, just for the sake of the experience or to see how it will go, anyhow. The owner came to me wanting to know if I could not amputate the cow's uterus. He said he had replaced it and sewed her up several times, but it would not stay. I went with him, and started in to exhibit my superior knowledge and skill; I ligated, as I thought, sufficiently tight and started to amputate with the écraseur. I probably cut too close to my ligature, and it slipped, and, perhaps, I used the écraseur too rapidly; but, anyhow, the blood came in the thickest stream I ever saw, and the cow promptly died. Fortunately, the man had the cow insured, and felt as little inclined to talk about it as I did. However, I would not now hesitate, with hope of more success, to amputate a uterus where I thought it was really indicated, where I am sure all other methods had failed or it had become gangrenous or much lacerated.

